# A Case Series of Pseudohyperkalemia: A Diagnostic Dilemma in Cancer Patients With Reactive Thrombocytosis

**DOI:** 10.7759/cureus.81851

**Published:** 2025-04-07

**Authors:** Ashwini More, Prafulla Parikh, Sujeet Kamtalwar, Avinash Pagdhune

**Affiliations:** 1 General Medicine, Advanced Centre for Treatment, Research and Education in Cancer (ACTREC) Tata Memorial Centre, Navi Mumbai, IND; 2 Biochemistry, Advanced Centre for Treatment, Research and Education in Cancer (ACTREC) Tata Memorial Centre, Navi Mumbai, IND

**Keywords:** essential thrombocytosis, infection, malignancy, pseudohyperkalemia, reactive thrombocytosis

## Abstract

Hyperkalemia is a life-threatening condition if not treated urgently. However, certain conditions can cause pseudohyperkalemia and the treating physician must be able to distinguish between the two to prevent complications of overtreatment that can lead to hypokalemia, itself a life-threatening condition. One of the causes of pseudohyperkalemia is thrombocytosis. Here we present three cases of pseudohyperkalemia secondary to reactive thrombocytosis in cancer (solid malignancy) patients. This case series emphasizes the importance of recognizing pseudohyperkalemia from a physician’s perspective to avoid inadvertent treatment.

## Introduction

Cancer patients face numerous triggers for hematologic and metabolic disturbances, amplifying the risk of diagnostic errors like pseudohyperkalemia. One such trigger is reactive thrombocytosis, which can be multifactorial [1]. Case studies in non-malignant patients have documented the occurrence of a single risk factor for reactive thrombocytosis in individual cases [1-3]. Still, studies documenting the presence of multiple risk factors in post-operative settings for solid malignancy patients are not available. This is the first case series from India that demonstrates that apart from solid malignancy, multiple risk factors (post-surgery acute blood loss, infection and splenectomy), double the chances of reactive thrombocytosis causing pseudohyperkalemia. The treating clinician must be aware of it. This case series aims to highlight such clinical scenarios.

## Case presentation

Case 1

A 29-year-old male underwent surgery (segmental hemi mandibulectomy) for carcinoma buccal mucosa at our center. After four days, he underwent re-exploration because of complications. He developed a surgical site infection, for which he was started on antibiotics (cefoperazone and sulbactam combination) as per the sensitivity report. On postoperative day 12, he was found to have high serum potassium (5.5 mEq/L). His other laboratory parameters were haemoglobin (Hb) 7.3 g/dL, total leukocyte count (TLC) 15,700/mm3, and platelet count 1,838,000/mm3 (Table 1). An electrocardiogram (ECG) did not reveal any signs of hyperkalemia. Preoperative complete blood count (CBC) and serum potassium were within normal limits. He was hemodynamically stable with normal systemic examination. He was started on treatment for hyperkalemia like salbutamol (10 mg) nebulization, glucose-insulin drip (10 U of regular insulin and 50 ml of dextrose 50%), low potassium diet (2 g/day), and potassium binders (sodium polystyrene sulfonate 15 g twice per day). However, hyperkalemia persisted (serum potassium 5.5 mEq/L). Considering the possibility of pseudohyperkalemia secondary to reactive thrombocytosis, the whole blood potassium level was advised, which was within normal limits (Table 1). Therefore, further treatment for hyperkalemia was discontinued, and he was discharged in stable condition.

**Table 1:** Summary of Important Laboratory Findings

Parameter	Reference range	Case 1	Case 2	Case 3
Haemoglobin (Hb, g/dL)	13 to 17	7.3	11.1	9.5
Total Leukocyte Count (TLC, /mm³)	4,000 to 10,000	15,700	12,500	13,970
Platelet Count (Platelets, /mm³)	150,000 to 400,000	1,838,000	1,118,000	814,000
Serum Potassium (mEq/L)	3.5 to 5.0	5.5	5.5	6.4
Whole blood Potassium (mEq/L)	3.5 to 5.0	4.2	4.3	4.7
Creatinine (mg/dL)	0.6 to 1.2	0.6	0.9	0.8

Case 2

A 72-year-old male, diagnosed with carcinoma penis, underwent partial penectomy and bilateral inguinal node dissection. He developed a surgical site infection. He was on antibiotics (piperacillin tazobactam) as per the sensitivity report. On postoperative day 15, he developed hyperkalemia (serum potassium was 5.5 mEq/L) (Table 1). He had a history of ischemic heart disease, for which he was on cardiac medications including angiotensin-converting enzyme inhibitor (ACEi). Because of hyperkalemia, ACEi was stopped. But hyperkalemia persisted. His CBC was Hb 11.1 g/dL, TLC 12,500/mm3, platelet 1,118,000/mm3 (Table 1). His pre-operative laboratory parameters were within normal limits. ECG was consistent with preoperative ECG and showed no signs of hyperkalemia. However, he was started on treatment for hyperkalemia given high risk due to cardiac comorbidity. His whole blood potassium was advised, which came to be normal (Table 1), and further treatment for hyperkalemia was stopped. Further admission course remained uneventful and he was discharged in stable condition.

Case 3

A 35-year-old male diagnosed with pseudomyxoma peritonei (mucinous adenocarcinoma) underwent cytoreductive surgery which needed splenectomy due to metastasis, followed by hyperthermic intraperitoneal chemotherapy (HIPEC). He developed hospital-acquired pneumonia and surgical site infection. He was already on antibiotics (cefoperazone and sulbactam combination). On postoperative day 10, the laboratory informed us of the critical value of serum potassium (6.4 mEq/L). His other laboratory parameters were Hb 9.5 g/dL, TLC 13,970/mm3, and platelet 814,000/mm3 (Table 1). The renal function test was normal. Pre-operative blood parameters were normal. He was hemodynamically stable and systemic examination was within normal limits. He was asymptomatic and ECG was normal. Due to severe hyperkalemia, he was also started on treatment. Due to reactive thrombocytosis, his whole blood potassium test was advised, which was normal (Table 1). Hence the diagnosis of pseudohyperkalemia was proven and further treatment for lowering serum potassium was stopped.

## Discussion

Hyperkalemia is defined as a serum potassium level of more than 5.0 mEq/L, however, these levels can vary as per different laboratory standards [4]. Commonly hyperkalemia is classified as mild (5-5.9 mEq/L), moderate (6-6.4 mEq/L), severe (>6.5 mEq/L) [5]. Pseudohyperkalemia is an artificially increased serum potassium that does not correlate with in vivo plasma potassium concentration [6]. Inadvertent treatment of pseudohyperkalemia may lead to hypokalemia, another life-threatening emergency. Hence, it is essential to differentiate between true and pseudohyperkalemia. Common causes of pseudohyperkalemia include: poor technique during collection and pre-analytical processing of blood samples (for example, fist clenching during venipuncture, vigorous mixing of blood sample tubes, in vitro hemolysis, sample exposed to low ambient temperature et cetera), and patient conditions that lead to pseudohyperkalemia (leukocytosis, thrombocytosis, inherited defects in erythrocyte membrane) [7]. Thrombocytosis can be divided into two groups: primary (essential) thrombocytosis and secondary (reactive) thrombocytosis [8]. Essential thrombocytosis is due to excessive platelet production by bone marrow, as seen in myeloproliferative neoplasm [9]. It is associated with an increased risk of thrombosis and bleeding compared to reactive thrombocytosis [9]. Reactive thrombocytosis can be due to acute blood loss, iron deficiency, acute or chronic infection, malignancy, asplenia, or chronic inflammatory diseases [1]. Reactive thrombocytosis resolves when the underlying causative condition is addressed [1]. Although reactive thrombocytosis is benign, the underlying cause (malignancy, infections, inflammation) can be associated with an increased risk of adverse outcomes. In our case series, all the patients had malignancy for which they underwent surgery. All of them had postoperative infections. While infections [1], malignancy [2], and acute blood loss [3] are the established causes of reactive thrombocytosis, we could not find any case studies in cancer patients (solid malignancy) documenting multifactorial reactive thrombocytosis and pseudohyperkalemia. One of the patients in our case series needed splenectomy due to metastasis, which is the cause of reactive thrombocytosis, which is similar to the case study in the literature by Sande et al. [10]. All patients were asymptomatic for hyperkalemia and ECG did not show any signs of hyperkalemia, which is one of the clues for diagnosis of pseudohyperkalemia [11]. Due to suspicion of pseudohyperkalemia secondary to reactive thrombocytosis, whole-blood potassium testing was advised for all the patients, which was normal, and further treatment to lower serum potassium was discontinued. Platelets release potassium during the clotting process, resulting in higher potassium concentrations in the serum as compared to plasma [12]. Simultaneous measurement of whole blood potassium (sample collected in the heparinized bulb) will help in diagnosing pseudohyperkalemia [12], where serum potassium will be increased but whole blood potassium will be normal. 

## Conclusions

Our case series highlights the importance of identifying pseudohyperkalemia promptly in patients with reactive thrombocytosis in the setting of malignancy, post-operative status with active infection and post-splenectomy. It will prevent iatrogenic hypokalemia, which can be life-threatening. Also, clinicians must have a high index of suspicion when the measured potassium is not correlating with baseline readings, or when the laboratory values and ECGs are not aligned with the clinical scenario. If pseudohyperkalemia is suspected, the concerned laboratory should be informed, and whole blood (heparinized sample) potassium levels need to be measured for correct diagnosis. 
